# Dynamics and Conformational Studies of TOAC Spin Labeled Analogues of Ctx(Ile^21^)-Ha Peptide from *Hypsiboas albopunctatus*

**DOI:** 10.1371/journal.pone.0060818

**Published:** 2013-04-09

**Authors:** Eduardo F. Vicente, Luis Guilherme M. Basso, Graziely F. Cespedes, Esteban N. Lorenzón, Mariana S. Castro, Maria José S. Mendes-Giannini, Antonio José Costa-Filho, Eduardo M. Cilli

**Affiliations:** 1 Departamento de Bioquímica e Tecnologia Química, Instituto de Química, UNESP-Univ Estadual Paulista, Araraquara/SP, Brazil; 2 Grupo de Biofísica Molecular Sérgio Mascarenhas, Instituto de Física de São Carlos, Universidade de São Paulo, São Carlos/SP, Brazil; 3 Brazilian Center for Protein Research, Department of Cell Biology, University of Brasília, Brasília/DF, Brazil; 4 Departamento de Análises Clínicas, Faculdade de Ciências Farmacêuticas, UNESP-Univ Estadual Paulista, Araraquara/SP, Brazil; 5 Departamento de Física, Faculdade de Filosofia, Ciências e Letras de Ribeirão Preto, Universidade de São Paulo, Ribeirão Preto/SP, Brazil; Centro Nacional de Biotecnologia - CSIC, Spain

## Abstract

Antimicrobial peptides (AMPs) isolated from several organisms have been receiving much attention due to some specific features that allow them to interact with, bind to, and disrupt cell membranes. The aim of this paper was to study the interactions between a membrane mimetic and the cationic AMP Ctx(Ile^21^)-Ha as well as analogues containing the paramagnetic amino acid 2,2,6,6-tetramethylpiperidine-1-oxyl-4-amino-4-carboxylic acid (TOAC) incorporated at residue positions n = 0, 2, and 13. Circular dichroism studies showed that the peptides, except for [TOAC^13^]Ctx(Ile^21^)-Ha, are unstructured in aqueous solution but acquire different amounts of α-helical secondary structure in the presence of trifluorethanol and lysophosphocholine micelles. Fluorescence experiments indicated that all peptides were able to interact with LPC micelles. In addition, Ctx(Ile^21^)-Ha and [TOAC^13^]Ctx(Ile^21^)-Ha peptides presented similar water accessibility for the Trp residue located near the N-terminal sequence. Electron spin resonance experiments showed two spectral components for [TOAC^0^]Ctx(Ile^21^)-Ha, which are most likely due to two membrane-bound peptide conformations. In contrast, TOAC^2^ and TOAC^13^ derivatives presented a single spectral component corresponding to a strong immobilization of the probe. Thus, our findings allowed the description of the peptide topology in the membrane mimetic, where the N-terminal region is in dynamic equilibrium between an ordered, membrane-bound conformation and a disordered, mobile conformation; position 2 is most likely situated in the lipid polar head group region, and residue 13 is fully inserted into the hydrophobic core of the membrane.

## Introduction

The fight against bacterial infections has become a major public health problem. Classical antibiotics commercialized nowadays are not always an efficient therapy due to the development of bacterial resistance [1]. Therefore, the search for new molecules from different sources, such as microbes, plants, amphibians, insects and mammals, is an interesting alternative strategy. In this context, cationic antimicrobial peptides (cAMPs) [2], [3] are promising. cAMPs are characterized by the high occurrence of basic amino acids, a considerable percentage of hydrophobic amino acids and a greater tendency to adopt amphipathic α-helical structures. Unlike classical antibiotics, which attack specific enzymes or receptors in the cell [4], cAMPs change membrane permeability and promote perturbation in the pathogen cell membrane, which is the basis of their mechanism of action [5].

Recently, Castro’s group has isolated a cationic antimicrobial peptide from the skin secretion of an arboreal South American frog, *Hypsiboas albopunctatus* [6]. This peptide – called Ctx(Ile^21^)-Ha (ceratotoxin-like peptide from *Hypsiboas albopunctatus*) – presents the following primary structure: Gly-Trp-Leu-Asp-Val-Ala-Lys-Lys-Ile-Gly-Lys-Ala-Ala-Phe-Asn-Val-Ala-Lys-Asn-Phe-(Ile/Leu) [7]. Interestingly, this sequence does not have similarity with other sequences found in amphibians, but it does have homology with the ceratotoxins peptide family, specifically ceratotoxin A [8]. The ceratotoxin peptide family exhibits biological activity against *E. coli* and other Gram negative bacteria, permeabilizing membranes and forming pores by a “barrel stave” mechanism [9]. Ctx(Ile^21^)-Ha showed biological activity against bacteria and fungi, including *Candida* [8]. This fungal species is involved in a variety of processes, such as mucocutaneous illnesses and invasive processes. Thus, due to the increase of the resistance to conventional drugs, analogues of this peptide could be used as new candidates for antimicrobial therapy, which requires the elucidation of its mode of action.

Spin labels have proven to be powerful tools for probing peptide structure and allowing the detailed study of the dynamics of these molecules. Although there are several approaches that can be used to covalently attach spin labels to peptides, our group has focused on the use of the paramagnetic amino acid TOAC (2,2,6,6-tetramethylpiperidine-1-oxyl-4-amino-4-carboxylic acid) [10]–[15]. This paramagnetic probe can be directly incorporated in the backbone of synthetic peptides, thus giving information about the orientation and dynamics of the peptide main chain. The procedure for attachment of the TOAC probe was introduced by Nakaie *et al.* [16], where the spin label was bound only to the peptide N-terminus. Since the synthesis of the Fmoc-TOAC by Marchetto *et al.* [17], however, it is possible to insert the paramagnetic amino acid in other positions of the peptide main chain [18]. Moreover, the use of TOAC allows one to evaluate, through ESR spectroscopy, the mobility of the spin-labeled peptide backbone inside the resin beads, thus helping to optimize the solid-phase peptide synthesis [19]–[23]. Previous studies have demonstrated that insertion of TOAC in a peptide sequence induces turns and helical structures due to its similarity with α-aminoisobutiric amino acid (Aib) [24]–[26].

In this paper, we used ESR along with other spectroscopies, such as fluorescence and circular dichroism, to investigate the interaction of the cAMP Ctx(Ile^21^)-Ha with a membrane mimetic environment. To do so, analogues containing the TOAC spin label in strategically determined positions were synthesized. These modifications were designed for an evaluation of the dynamics and conformational properties of the spin label, thus providing information about the peptide topology in LPC micelles.

## Materials and Methods

### Chemicals and Microorganisms

Analytical grade reagents from commercial suppliers were used in this work and all solutions were prepared with ultrapure water (Barnstead/Thermolyne-E-pure, Dubuque, IA, USA). Solvents for chromatographic procedures were HPLC grade (Tedia, Fairfield, OH, USA). All natural 9-fluorenylmethyloxycarbonyl (Fmoc) amino acids and Rink-amide MBHAR resin were purchased from SynBioSci (Livermore, CA, USA) and Novabiochem (Darmstadt, Germany). Solvents and reagents for peptide synthesis were acquired from Sigma-Aldrich Co. (St. Louis, MO, USA), Fluka (St. Louis, MO, USA) and J. T. Baker Chemical Co. (Center Valley, PA, USA). The detergent 1-palmitoyl-2-hydroxy-*sn*-glycero-3-phosphocholine (LPC) used in micelles was purchased from Avanti Polar Lipids. The bacterial strains *Escherichia coli* (ATCC 25922), *Staphylococcus aureus* (ATCC 25923), *Pseudomonas aeruginosa* (ATCC 27853) and *Bacillus subtilis* (ATCC 19659) were obtained from the “Banco de Culturas Tropicais - Fundação André Tosello” (Campinas, SP, Brazil). *Candida albicans* (ATCC 90028) and *Cryptococcus neoformans* (ATCC 90012) were originally obtained from the Mycology Laboratory of the Department of Clinical Analysis at Faculdade de Ciências Farmacêuticas, Universidade Estadual Paulista (Araraquara, SP, Brazil).

### Peptide Synthesis

The Ctx(Ile^21^)-Ha peptide and its analogues containing the TOAC spin label (with amidated C-terminus) were manually synthesized according to the standard N^α^-Fmoc protecting group strategy [27]. The side chain protecting group Boc (*t*-butoxycarbonyl) was used for the Fmoc-amino acids Lys and Trp, while Trt (Trityl) and tBu (t-Butyl) were applied for Asn and Asp, respectively. After coupling of the C-terminal amino acid to Rink-amide-MBHAR, the α-amino group deprotection step was performed in 20% piperidine/dimethylformamide (DMF) for 1 and 20 min. The amino acids were coupled at three fold excess using N,N’-diisopropylcarbodiimide (DIC)/N-hydroxybenzotriazole (HOBt) in 50% (v/v) DCM (methylene chloride)/DMF or, when a recoupling was needed, 2-(1H-benzotriazole-1-yl)-1,1,3,3-tetramethyluronium-hexafluorophosphate (TBTU)/diisopropylethylamine (DIEA) in 50% (v/v) DCM/*N*-methylpyrrolidone (NMP). After 2 hours of coupling, the ninhydrin test was performed to monitor the completeness of the reaction. For the TOAC coupling, we used 1-[bis(dimethylamino)methylene]-1H-1,2,3-triazolo-[4,5-b]pyridiniumhexafluorophosphate-3-oxide] (HATU) /DIEA and spin label amino acid with 3.0, 4.0 and 1.2 molar equivalent excess over the amino component in the resin, respectively. The coupling of the next amino acid required different conditions to reach satisfying results. In this coupling, the temperature was 60°C and the molar excess was 5.0 for Fmoc-Ala to [TOAC^13^]Ctx(Ile^21^)-Ha and Fmoc-Gly to [TOAC^2^]Ctx(Ile^21^)-Ha, using HATU/DIEA as acylating reagents, in constant stirring, for 2 h. The yield of coupling was monitored by HPLC after a cleavage reaction of 20 mg of the peptidyl-resin collected immediately after the first, third and sixth coupling steps.

For all peptides, cleavage from the resin and removal of the side chain protecting groups were simultaneously performed with 90% trifluoracetic acid (TFA), 5% triisopropylsylane (TIS) and 5% Milli-Q water for 2 hours. After this procedure, the crude peptides were precipitated with anhydrous ethyl ether, separated from soluble non-peptide material by centrifugation, extracted into a 30% acetonitrile/H_2_O solution (v/v) and lyophilized.

After cleavage, the extracted spin-labeled analogues were submitted to alkaline treatment for complete reversion (monitored by analytical HPLC) of the N−O protonation that occurs during the acid cleavage [10]. After this procedure, purification was performed by semi-preparative HPLC Beckman System Gold (Brea, CA, USA) with a reverse phase C-18 column in a linear gradient, using aqueous 0.02 mol L^−1^ ammonium acetate (pH 5.0) and 90% acetonitrile in ammonium acetate solution as solvents A and B, respectively. The flow rate was 5 mL/min. The peptide homogeneity was checked by analytical HPLC Varian (Santa Clara, CA, USA), using solvents A (0.045% TFA:H_2_O) and B (0.036% TFA:ACN) with a linear gradient of 5–95% (v/v) of solvent B for 30 min, at a flow rate of 1.0 mL/min and UV detection at 220 nm. The presence of the peptide was confirmed by Electrospray Mass Spectrometry on a ZMD Micromass model apparatus (Milford, MA, USA) and amino acid analysis (Shimadzu Corp. model LC-10A/C-47A, Kyoto, Japan).

### Hemolysis and Antimicrobial Assays

These assays were performed according to the experimental procedure described by Castro *et al.* [6].

### Circular Dichroism Studies

Circular dichroism (CD) spectra were recorded at 25°C on a Jasco Products Company, Inc. (Oklahoma City, OK, USA) J-715 CD spectropolarimeter using a 1 mm path-length quartz cell. Samples containing 80 µmol L^−1^ of peptides dissolved in Milli-Q water, 60% of trifluoroethanol (TFE) in 10 mmol L^−1^ Tris, 150 mmol L^−1^ NaCl, pH 7.4 buffer solution (v/v) or 10 mmol L^−1^ LPC micelles were prepared. Trifluorethanol (TFE) and LPC were used to mimic membrane environments. Spectra were acquired every 0.5 nm from 250 to 194 nm at a scan speed of 50 nm min^−1^, with a 2 nm bandwidth and a response time of 3 s. Each spectrum represents an average of 16 successive scans and is expressed as molar ellipticity [θ] (deg. cm^2^. dmol^−1^) [28].

### Steady State Fluorescence Studies and Quenching by Acrylamide

All fluorescence experiments were performed on a Cary Eclipse Varian (Santa Clara, CA, USA) spectrofluorimeter with an excitation wavelength of 280 nm. Emission spectra were recorded between 300 and 500 nm. Measurements were carried out in 10 mmol L^−1^ Tris, 150 mmol L^−1^ NaCl, pH 7.4 and at 25°C. Interaction of the peptides with LPC micelles was monitored by the fluorescence enhancement of tryptophan by titration of LPC micelles to the peptide samples. Peptides and final LPC concentrations were, respectively, 10 µmol L^−1^ and 10 mmol L^−1^. Fluorescence quenching studies were carried out by titration of acrylamide from a 4 mol L^−1^ stock solution to the final concentration 0.05 mol L^−1^ with and without 10 mmol L^−1^ LPC micelles. The peptide/lipid molar ratio was 1∶1,000. Quenching constants *K*_SV_ were determined by linear regression with the Stern-Volmer equation:

F_0_/F = 1 + *K*_SV_ × [Q].where F_0_ and F represent the fluorescence intensities in the absence and in the presence of acrylamide, respectively, and [Q] is the total molar concentration of the quencher in the sample.

### ESR Studies

ESR experiments were performed on a Varian (Santa Clara, CA, USA) E-109 X-band (9.5 GHz) CW-EPR spectrometer at room temperature (22°C) using ﬂat quartz cells. The experimental acquisition parameters were: center field, 3362 G; sweep width, 100 G; modulation amplitude, 0.5 G; modulation frequency, 100 kHz; microwave power, 10 mW; time constant, 128 ms, and acquisition time, 150 s.

Nonlinear least-squares simulations of the ESR spectra of the TOAC-containing peptides were carried out by using either the NLSL program developed by Freed and co-workers [29], [30] or the Multicomponent LabView software written by Christian Altenbach [31].

**Figure 1:**
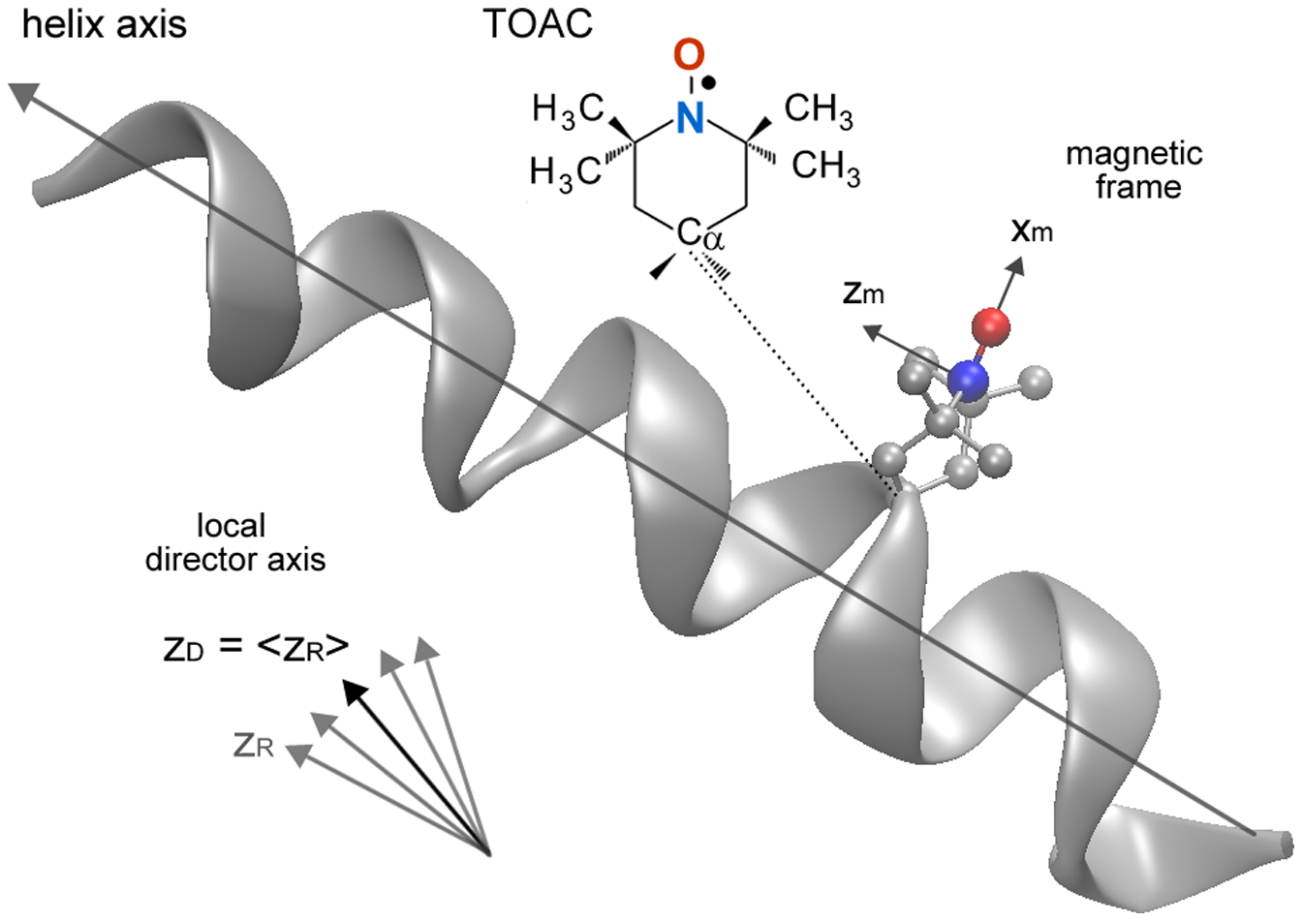
Definition of the principal magnetic axes (*x_m_*, *y_m_*, *z_m_*) oriented relative to the nitroxide molecular frame in a TOAC-labeled α-helical peptide. The y_m_ axis is perpendicular to the others, forming a right-handed coordinate system. The rotational diffusion axes (*x_R_*, *y_R_*, *z_R_*) are taken to coincide with the magnetic frame (see text). The local director, *z_D_*, is defined as the average orientation of the *z_R_* axis over the course of its motion. In the rotational diffusion frame, where *z_R_* is fixed, *z_D_* traces a trajectory around *z_R_*. The orienting potential is then expressed as a function of the polar angles 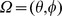 of *z_D_* in the rotational diffusion frame. The TOAC spin label was inserted at position 13 according to Ghimire and collaborators [32] using MolMol program [33].

The Brownian dynamics of the TOAC-labeled peptides were analyzed using different models for the rotational diffusion tensor: isotropic rotation, axial rotation, or the fully anisotropic model. Since TOAC adopts a twisted boat geometry, it is characterized by only one degree of freedom, the flip of its six-membered ring. However, when this spin label is incorporated into a helix, the conformation placing the nitroxide z-axis almost parallel to the α-helix axis is the most common (Figure 1) [34]. Thus, the rotational diffusion axes were initially taken to coincide with the magnetic frame, in which the g and A tensors are defined: the *x_m_* axis points along the N−O bond direction, the *z_m_* axis lies along the axis of the 2*p_z_* orbital of the nitrogen, and the *y_m_* axis is perpendicular to the others (Figure 1). During the simulation process, though, the diffusion tilt angles 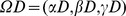, which are the Euler angles of the magnetic axes in the rotational diffusion frame, were allowed to vary, but no better fit was obtained, so they were kept null.

Additionally, the ESR spectra of the membrane-bound peptides were analyzed with the microscopic order with macroscopic disorder (MOMD) model [35], which takes into account the tendency of the spin probe to become partially ordered with respect to a local director that is itself randomly oriented in the sample. The microscopic molecular ordering of the spin label is characterized by the order parameter *S*_0_, defined as
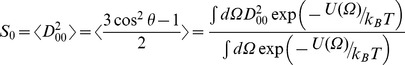
which reflects the restricted range of orientations of the spin probe imposed by the orienting potential



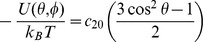



In the above equations, *k_B_* is the Boltzmann’s constant, *T* is the temperature, 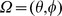 are the polar angles of the local director in the rotational diffusion axis frame (Figure 1), and the dimensionless coefficient 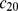
is the parameter used in the fitting process. The final theoretical MOMD spectrum is then calculated by integration over the distribution of the local director orientations. In our simulations, 30 orientations were needed for convergence.

Seed values for the magnetic g-tensor components (*g_xx_*, *g_yy_*, *g_zz_*) were taken from Nesmelov *et. al* [36]. However, since the nitroxide g-tensor values strongly depend on the environmental polarity and hydrogen bonding and can only be determined with high accuracy using high-field ESR spectroscopy [37], [38], we allowed the components to vary slightly during the fitting process. On the other hand, changes in *A_zz_* due to solute-solvent interactions can be obtained with relative accuracy from the X-band ESR spectra of frozen samples. So, the procedure to determine starting values for the magnetic hyperfine-tensor elements (*A_xx_*, *A_yy_*, *A_zz_*) was the following: from the 12 K ESR spectra, we estimated the *A_zz_* value by measuring the maximum hyperfine splitting. Besides that, the isotropic hyperfine splitting (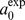) was calculated from the spectrum obtained at high temperatures (> 50°C) as one-half the distance between the low- and high-field lines. Assuming initially an axial symmetry for the A-tensor 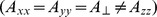 and using 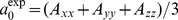, we were able to estimate the starting values for the TOAC 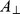 as 5.8 G for both [TOAC^0^]Ctx(Ile^21^)-Ha and [TOAC^2^]Ctx(Ile^21^)-Ha and 6.5 G for [TOAC^13^]Ctx(Ile^21^)-Ha in buffer solution, and 6.2 G for TOAC^0^ and 5.5 G for both TOAC^2^ and TOAC^13^ derivatives in LPC micelles. The uncertainty found was 0.8 G for all the A-tensor component values.

During the simulation process, the g- and A-tensor seed values were kept constant until a good fit was achieved. This was done so that each one of the other parameters was varied independently thus avoiding high correlations between them. After that, the g- and A-tensor components were again allowed to vary slightly until the best theoretical spectrum was achieved. Finally, different sets of seed values for the TOAC diffusion parameters were used in order to avoid local minima and to estimate the uncertainty for each parameter.

## Results and Discussion

### Peptide Synthesis

**Figure 2:**
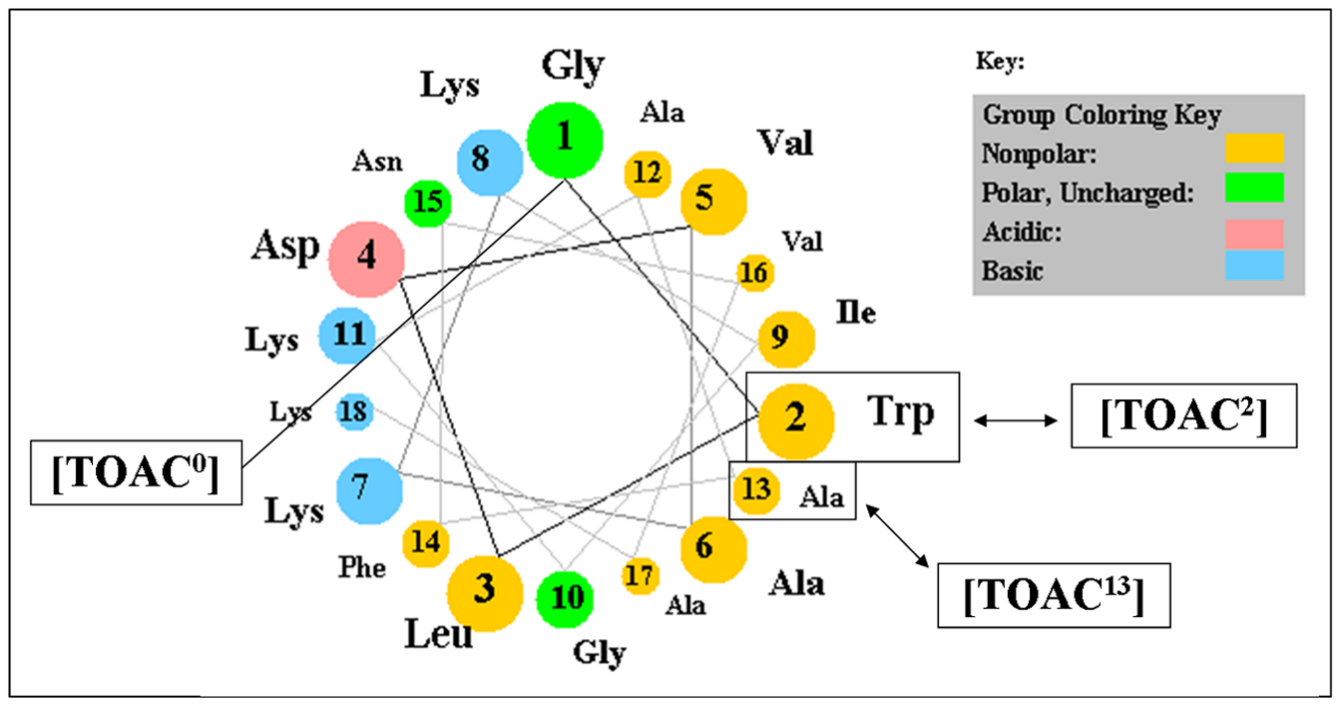
A Schiffer–Edmundson helical wheel of the peptide Ctx(Ile^21^)-Ha. The modifications/additions of the paramagnetic amino acid TOAC are in the square highlighted.

We have used the solid-phase peptide synthesis and the TOAC spin probe to assess the structural dynamics of Ctx(Ile^21^)-Ha. This peptide has shown antimicrobial activity against Gram positive and Gram negative bacteria and fungi. In addition, Ctx(Ile^21^)-Ha has shown high amounts of α-helical structure in the presence of TFE and LPC [8]. In this study, three analogues containing the paramagnetic amino acid TOAC strategically inserted in different positions of the sequence (Table I) were designed. Ctx(Ile^21^)-Ha acquires an amphipathic α-helix structure due to the amino acid distribution around the helix, producing hydrophobic and hydrophilic faces as represented by a Schiffer-Edmundson α-helix wheel projection [39] (Figure 2). The first analogue [TOAC^13^]Ctx(Ile^21^)-Ha has the TOAC spin label in a central point of the apolar face, where it replaces alanine at position 13. In the second peptide, called [TOAC^2^]Ctx(Ile^21^)-Ha, TOAC was introduced in the N-terminal portion by replacing the tryptophan at the second position of the backbone. The last analogue was obtained by adding TOAC at the N-terminus - [TOAC^0^]Ctx(Ile^21^)-Ha. The N-terminal region was studied because of its importance for the biological activity, pore formation of hemolytic peptides [40] and biological selectivity of AMPs [41]. Furthermore, Lopes and collaborators [42] demonstrated the importance of the N-terminal position of the synthetic antimicrobial peptide analog of Plantaricin 149 on membrane disruption. Our peptide analogues were then designed to probe three different positions of their structures with emphasis on the N-terminus.

**Table 1:** Amino acid sequences and characterization features of the synthetic peptides.

Peptide	Sequence^a^	RetentionTime (min)^b^	M_r_(g mol^−1^)	Purity (%)
Ctx(Ile^21^)-Ha	GWLDVAKKIGKAAFNVAKNFI	18.270	2,289.7	96
[TOAC^0^]Ctx(Ile^21^)-Ha	**O**GWLDVAKKIGKAAFNVAKNFI	18.611	2,486.0	97
[TOAC^2^]Ctx(Ile^21^)-Ha	G**O**LDVAKKIGKAAFNVAKNFI	18.494	2,299.8	98
[TOAC^13^]Ctx(Ile^21^)-Ha	GWLDVAKKIGKA**O**FNVAKNFI	21.038	2,414.9	96

a The letter (**O**) represents the paramagnetic amino acid TOAC.

b Values obtained from analytical RP-HPLC with C^18^ column and program: 5–95 % B in 30 min. Solvent A: 0.045% TFA: H_2_O and B: 0.036% TFA: ACN. The peptides were eluted at 1.0 mL min^−1^ of flow rate and detected at 220 nm.

Standard protocols were used for solid-phase peptide synthesis, except for the coupling of the amino acid after the TOAC incorporation. The p*K*_a_ of the TOAC ammonium group is low, approximately ∼ 5.5 [16], which directly affects the nitrogen nucleophilicity, making difficult the attack to the next Fmoc-amino acid carbonyl group. Therefore, this procedure requires more efficient methods and reagents to obtain high yield of synthesis [43] (see Materials and Methods for details). The six cycles of coupling using high temperature and more effective coupling activators, as HATU, were not enough to reach 100% of yield. In the synthesis of [TOAC^2^]Ctx(Ile^21^)-Ha and [TOAC^13^]Ctx(Ile^21^)-Ha only 60% of product was obtained. Despite this problem, the TOAC-peptides were successfully obtained using the solid-phase peptide synthesis methodology. After the cleavage of the analogues, the crude spin labeled peptides were submitted to alkaline treatment (pH 10, 2 h, 25°C) to reverse the N−O protonation that occurred during the TFA cleavage of the peptide [10]. The overall results of the peptide syntheses are shown in Table I. The purity achieved after HPLC purification was higher than 95%.

### Biological Activity

To investigate the effects of TOAC addition on the biological activity of Ctx(Ile^21^)-Ha, antimicrobial and hemolytic assays were carried out (Table II). Antibacterial activities were evaluated *in vitro* against the Gram negative bacterial strains *E. coli* and *P. aeruginosa*, and Gram positive *S. aureus* and *B. subtilis*. The fungi tested were *Candida albicans* and *Cryptococcus neoformans*. All these microorganisms are involved in several human pathologies, such as hospital and urinary tract infections, fungal meningitis and general gastroenteritis, often observed in immunosuppressed patients [44]. Hemolytic activity using human erythrocytes was also evaluated in order to measure the toxicity of the peptides in higher eukaryotic cells (Table II).

**Table 2:** Minimal Inhibitory Concentrations (MICs) against microorganisms and concentration producing 50% hemolysis of human erythrocytes (HC_50_) of the peptides.

			Bacteria											
		*Gram* (−) *Gram* (*+*)								Fungus					
		*E.* *coli*		*P.* *aeruginosa*		*S. aureus*		*B.* *subtilis*		*Candida* *albicans*		*Cryptococcus neoformans*		Humanerythrocytes	
Peptide		MIC (µmol L^−1^)								MIC (µmol L^−1^)				HC_50_(µmol L^−1^)	
Ctx(Ile^21^)-Ha	8		8		2		1		31.3		62.5		7.1		
[TOAC^0^]Ctx(Ile^21^)-Ha	16		64		8		2		62.5		62.5		2.6		
[TOAC^2^]Ctx(Ile^21^)-Ha	8		32		8		1		31.3		62.5		19.0		
[TOAC^13^]Ctx(Ile^21^)-Ha	8		16		8		2		7.8		31.3		1.1		

***Observation:*** MIC values are reported as the mode of three independent assays.

The results showed that all analogues of Ctx(Ile^21^)-Ha had biological activity against Gram positive and Gram negative bacteria and fungi. In addition, the data showed that the peptides exhibited a decreased antibacterial activity against Gram negative bacteria when TOAC is close to the N-terminus. [TOAC^13^]Ctx(Ile^21^)-Ha showed similar antibacterial activity in Gram negative bacteria when compared with Ctx(Ile^21^)-Ha; while in *S. aureus*, the activity was four times lower. The analogue [TOAC^0^]Ctx(Ile^21^)-Ha presented the lowest activity in Gram negative bacteria, but kept its activity against Gram positive bacteria. These findings demonstrate that the N-terminal group is indeed important for the selectivity of AMPs as found by Crusca et al. [41].

The antifungal and hemolytic activities of the analogue [TOAC^13^]Ctx(Ile^21^)-Ha were higher than those observed for Ctx(Ile^21^)-Ha. The TOAC structure induces an increase in the helical content of the peptide, which could explain the increase of the activity against fungi and human erythrocytes [45]. The peptide [TOAC^2^]Ctx(Ile^21^)-Ha showed the lowest hemolytic activity. Lopes and collaborators [42] have shown that the interaction of the N-terminal region with the membrane is the first step in the pore formation. The incorporation of TOAC near the N-terminal region causes a higher rigidity in this segment of the peptide, which may affect the mechanism of action in this type of membrane, thus decreasing the hemolytic activity [46]. In addition, the differences between the antibacterial and hemolytic activities can be due to the differences between the prokaryotic and eukaryotic membrane compositions. The different composition of the membranes could promote different modes of action of the peptides [47]. Finally, the lower antifungal activity of [TOAC^0^]Ctx(Ile^21^)-Ha against *Candida albicans* can again be explained by the presence of the TOAC at the N-terminus of this peptide. This large and hydrophobic spin labeled amino acid may not support the interaction between the peptide and the cell wall and thus decreases the antifungal activity [7] .

### Circular Dichroism Studies

**Figure 3:**
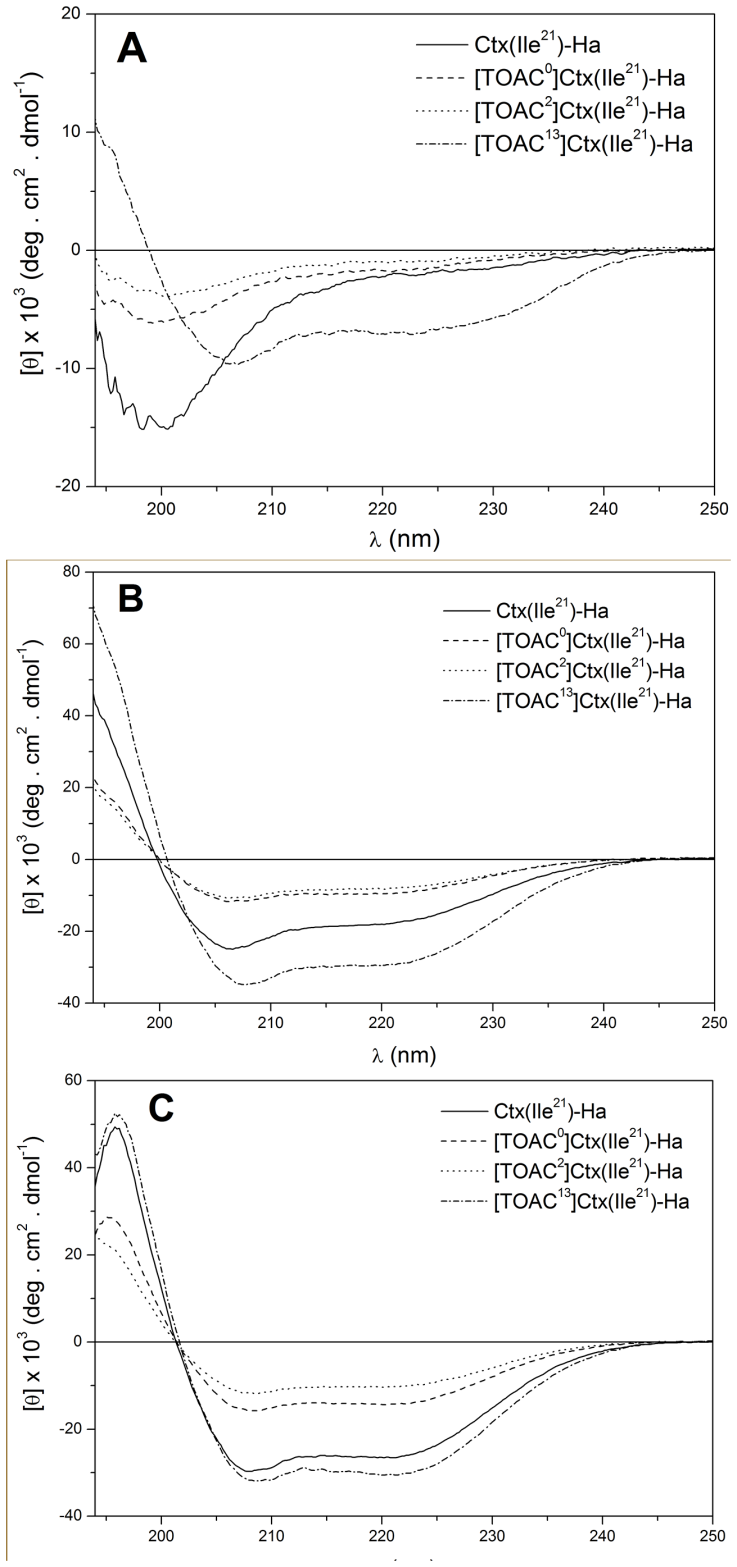
CD spectra of synthetic peptides. The spectra were obtained in aqueous solution (**A**), in the presence of 60% (v/v) trifluoroethanol (**B**), and in the presence of LPC 10 mmol L^−1^ (**C**). The peptide concentration was 80 µmol L^−1^.

Secondary structure measurements were performed by CD spectroscopy in aqueous solution, 60% TFE/buffer solution (v/v), and 10 mmol L^−1^ of the membrane mimetic LPC in order to obtain information about the structures of these peptides. Figure 3 shows the far-UV CD spectra of the peptides. In aqueous solution, Ctx(Ile^21^)-Ha, [TOAC^0^]Ctx(Ile^21^)-Ha and [TOAC^2^]Ctx(Ile^21^)-Ha displayed a typical spectrum of a disordered structure (Figure 3A) most likely due to hydrogen bonding between peptide bonds and water molecules. On the other hand, the [TOAC^13^]Ctx(Ile^21^)-Ha CD spectrum in water showed three bands, one positive at 196 nm and two negatives at 208 and 222 nm, typical of an α-helical secondary structure. This structural change can be explained by the TOAC incorporation in the central part of the peptide sequence, since this amino acid spin label is a strong structure inducer [48]. The addition of TFE, a well-known secondary structure inducer [49], and LPC micelles promoted conformational changes on all peptides, but at different degrees (Figures 3B and 3C). The spectra displayed the typical features for α-helical structures, with the following order of helicity as determined according to Chen and collaborators [50]: [TOAC^13^]Ctx(Ile^21^)-Ha > Ctx(Ile^21^)-Ha > [TOAC^0^]Ctx(Ile^21^)-Ha > [TOAC^2^]Ctx(Ile^21^)-Ha in both TFE and LPC micelle conditions.

The CD studies and the antimicrobial and hemolytic assays indicate that there is a relationship between the degree of helicity and the biological activity of these antimicrobial peptides. For instance, [TOAC^2^]Ctx(Ile^21^)-Ha and [TOAC^0^]Ctx(Ile^21^)-Ha exhibited the least amount of α-helix and the lowest biological activities, presumably due to the rigidity caused by the TOAC incorporation. Also, this result strongly indicates that the N-terminal portion is somehow involved in the biological activity of this peptide. These results are also in agreement with other studies, which suggest that the α-helical structure adopted by the peptides is an important key for the maintenance and specificity of their biological activities: the higher the α-helicity of AMPs, the greater the hemolytic activity [51], [52].

### Fluorescence Studies

**Figure 4:**
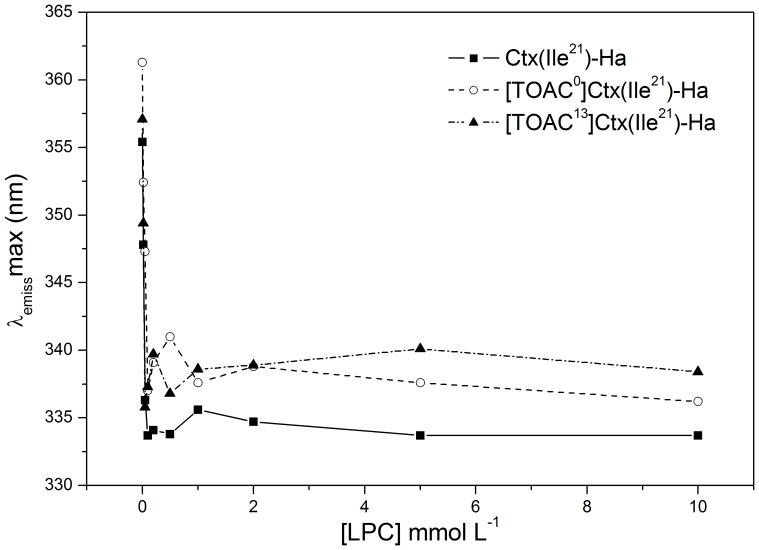
Fluorescence emission maxima of the synthetic peptides as a function of the concentration of LPC micelles in TRIS buffer pH 7.4 at 25°C. The concentration of the peptide was 10 µmol L^−1^.

**Figure 5:**
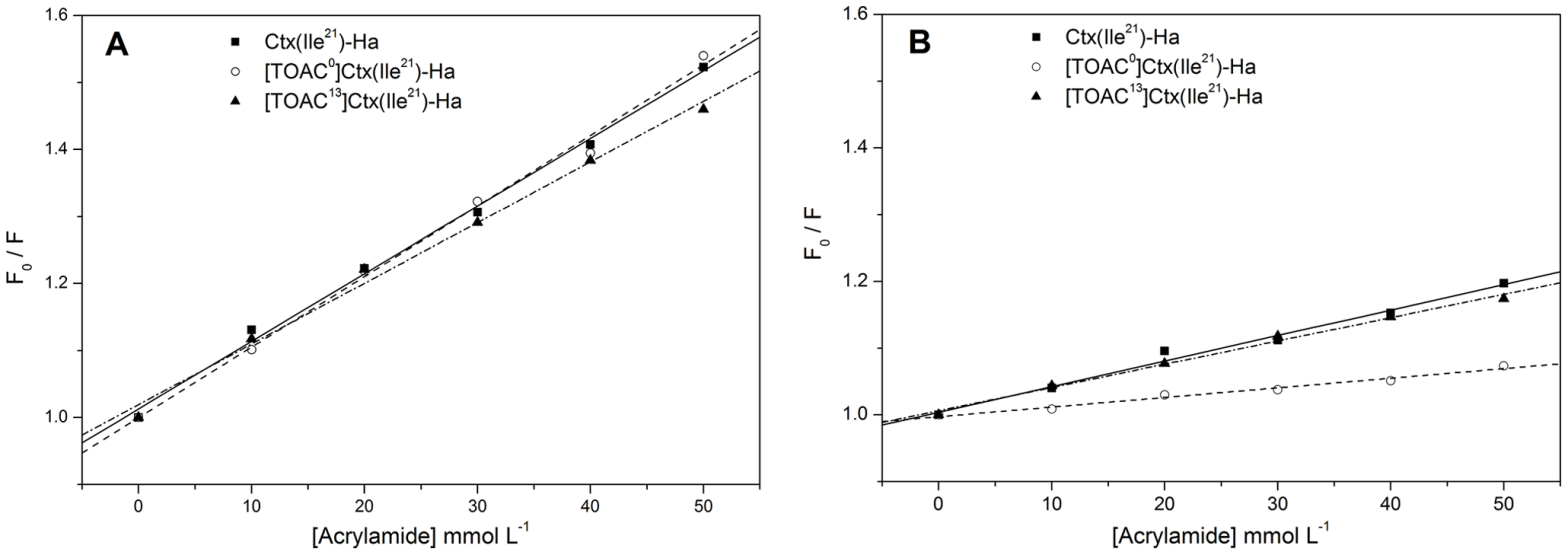
Variation of the Trp fluorescence for the peptides Ctx(Ile^21^)-Ha, [TOAC^0^]Ctx(Ile^21^)-Ha and [TOAC^13^]Ctx(Ile^21^)-Ha in aqueous solution (A) and in LPC micelles (B), in presence of acrylamide at different concentrations.

To further examine the effects of LPC micelles on the insertion of the peptides into such a membrane mimetic environment, we analyzed the fluorescence emission spectra of the Trp residues. Except for the analogue [TOAC^2^]Ctx(Ile^21^)-Ha, all peptides have a Trp residue at position 2 of their sequences. In aqueous solution, the wavelength of maximum emission (λ_max_) is approximately 357 nm (Table III), similar to *N*-acetyl-L-tryptophanamide (NATA) in water [53], indicating that the peptides do not aggregate in this case. Addition of LPC to the peptide solutions, however, causes a blue shift in the emission spectra. The λ_max_ values decreased to about 335 nm, which indicates that the probe is immersed in a hydrophobic environment (Figure 4). In addition, to gain insights on the membrane topology of the antimicrobial peptides, we investigated the accessibility of their Trp residues to the aqueous medium by using the water-soluble reagent acrylamide. The Stern-Volmer quenching constants (*K*_SV_) were calculated from linear regression of the maximum fluorescence intensity spectra upon acrylamide titration to free peptide solution (Figure 5A) and to the peptide immersed in LPC micelles (Figure 5B). As shown in Table III, the data reveal that all peptides in solution present a highly exposed Trp to water, with *K*_SV_ values around 10 L mol^−1^. A less efficient quenching in aqueous solution was observed for [TOAC^13^]Ctx(Ile^21^)-Ha (see Figure 5A and Table III), which indicates a less water-exposed Trp for this analogue compared to the other peptides. This result might be explained by the α-helical structure induced in the peptide after TOAC incorporation around residue 13. In the presence of LPC micelles, the *K*_SV_ values for all peptides were significantly decreased (Figure 5B, Table III), thus suggesting poor accessibility of the Trp residues to the aqueous phase. This is also consistent with binding and incorporation of the peptides into the membrane mimetic. Moreover, Ctx(Ile^21^)-Ha and [TOAC^13^]Ctx(Ile^21^)-Ha presented similar *K*_SV_ values, suggesting the same water-accessibility of their Trp residues. This result also indicates that substitution of an Ala residue at position 13 by the TOAC spin label does not affect the exposition of the N-terminal region to the aqueous phase, despite the structural changes caused by TOAC insertion. Finally, the lowest *K*_SV_ value presented by [TOAC^0^]Ctx(Ile^21^)-Ha in the presence of LPC micelles could be explained by an additional quenching mechanism by TOAC [54], most likely due to the peptide folding, which could bring TOAC and Trp residues closer together.

**Table 3:** Tryptophan fluorescence emission maxima and K_SV_ values for Ctx(Ile^21^)-Ha and analogues at 10 µmol L^−1^ in TRIS buffer solution or at 10 mmol L^−1^ of LPC micelles.

Peptide	LPC Micelles		Buffer Solution	
	λ_máx_	*K*_sv_L mol^−1^	λ_máx_	*K*_sv_L mol^−1^
Ctx(Ile^21^)-Ha	**334**	**3.8 ± 0.2**	**356**	**10.1 ± 0.4**
[TOAC^0^]Ctx(Ile^21^)-Ha	**335**	**1.4 ± 0.1**	**357**	**10.5 ± 0.4**
[TOAC^13^]Ctx(Ile^21^)-Ha	**339**	**3.5 ± 0.1**	**357**	**9.0 ± 0.3**

### ESR Measurements

ESR experiments were used to probe the structural dynamics of TOAC-containing Ctx(Ile^21^)-Ha analogues in aqueous solutions and in the presence of LPC micelles and also to gather information on the peptide topology in this membrane mimetic [55]. Because TOAC is rigidly coupled to the peptide, its ESR spectrum reflects the dynamics of the peptide backbone [20], [56]. Therefore, the correlation times and order parameters obtained from TOAC ESR spectra may help resolving distinct conformations of the peptide backbone. The use of TOAC in comparison with the well-known site-directed spin labeling (SDSL) methodology, in which the side chain of a native or an engineered cysteine is used to attach an ESR probe, such as the methanethiosulfonate spin label, presents the advantage of the lower TOAC flexibility. Despite the wide application of SDSL/ESR on the elucidation of macromolecular structure and conformational dynamics of biomolecules [57], [58], the intrinsic conformational flexibility of side chain-attached spin labels renders the analysis of backbone conformations more difficult.

**Figure 6:**
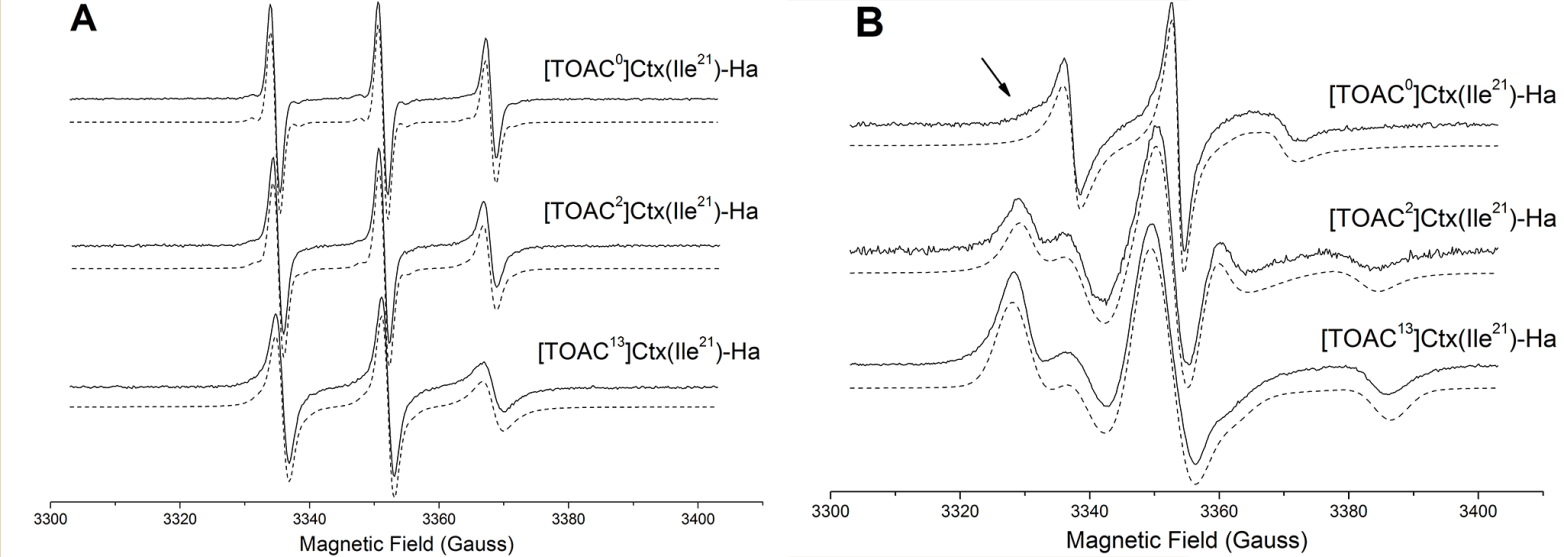
ESR spectra (solid lines) of the TOAC-labeled peptides and the best nonlinear least-squares fits (dashed lines) in aqueous solution (A) and LPC micelles (B) acquired at 22°C. The concentration of the peptide was 80 µmol L^−1^.

Figure 6 shows the ESR spectra of three different Ctx(Ile^21^)-Ha peptide analogues, [TOAC^0^]Ctx(Ile^21^)-Ha, [TOAC^2^]Ctx(Ile^21^)-Ha and [TOAC^13^]Ctx(Ile^21^)-Ha, in aqueous solution (Figure 6A) and in the presence of LPC micelles (Figure 6B) along with the best fits obtained from nonlinear least-squares simulations. The magnetic and dynamic parameters calculated from the spectra as described in Materials and Methods are displayed in Tables IV and V, respectively.

**Table 4:** Magnetic hyperfine-tensor components, given in Gauss units, for the TOAC-labeled Ctx(Ile^21^)-Ha analogues used in the nonlinear least-squares fits to the ESR spectra obtained at 22°C.

Peptide	Buffer Solution					LPC Micelles					
	*A_xx_*	*A_yy_*	*A_zz_*	*A_0_*	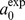	*A_xx_*	*A_yy_*	*A_zz_*	*A_0_*	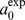	
[TOAC^0^]Ctx(Ile^21^)-Ha	6.0	6.0	38.00	16.67	16.60	6.07.5	6.06.5	37.8033.70	16.6015.90	16.38	
[TOAC^2^]Ctx(Ile^21^)-Ha	6.0	6.0	37.10	16.37	16.38	7.5	6.5	31.45	15.15	15.50	
[TOAC^13^]Ctx(Ile^21^)-Ha	6.0	6.0	37.00	16.33	16.30	7.5	6.5	30.10	14.70	15.05	

Observations.

•The components of magnetic g-tensor used in the nonlinear least-squares simulations for all the TOAC-containing peptides were: g_xx_ = 2.0096, g_yy_ = 2.0067 and g_zz_ = 2.0036.

•TOAC^0^ derivative incorporated into LPC micelles presented two spectral components. Populations: site 1 (40%), site 2 (60%). Uncertainty: 2%.

•The experimental isotropic hyperfine coupling 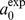 was calculated from the corresponding ESR spectra at 50°C.

•A_0_ was calculated as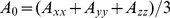 with the A-tensor values obtained from nonlinear least-squares simulations.

•Uncertainty of the A-tensor component values: A_xx_ (5%), A_yy_ (5%), A_zz_ (2%).

**Table 5:** Dynamics, structural and line width parameters obtained from nonlinear least-squares fits to the ESR spectra of TOAC-labeled Ctx(Ile^21^)-Ha analogues acquired at 22°C.

Peptide	R_x_ (τ_x_)	R_y_ (τ_y_)	R_z_ (τ_z_)	<τ>	Δ	*W_0_*	*c*_20_	*S*_0_
*Buffer*								
TOAC^0^	110.0 (0.15)	110.0 (0.15)	110.0 (0.15)	0.15	0.7	1.1	–	–
TOAC^2^	47.8 (0.35)	47.8 (0.35)	47.8 (0.35)	0.35	0.7	1.2	–	–
TOAC^13^	21.4 (0.78)	21.4 (0.78)	21.4 (0.78)	0.78	0.7	1.9	–	–
*LPC*								
TOAC^0^	16.2 (1.02)4.1 (4.1)	16.2 (1.02)6.5 (2.6)	29.5 (0.56)5.1 (3.3)	0.843.3	––	2.0	0.350.64	0.070.14
TOAC^2^	2.19 (7.7)	1.51 (10.9)	0.81 (20.6)	12.0	1.8	5.1	1.72	0.38
TOAC^13^	0.51 (32.7)	0.51 (32.7)	0.19 (85.5)	45.0	4.6	6.8	6.46	0.83
*Observations*•Δ *and W_0_ are, respectively, the calculated Gaussian line width and the experimental peak-to-peak width of the central line, and are given in Gauss units;*•*R-component and τ-component values are given in 10^7^ s* ^−*1*^ * and ns, respectively;*•*TOAC^0^ derivative incorporated into LPC micelles presented two components. Populations: site 1 (40%), site 2 (60%). Uncertainty: 2%.*•*Uncertainty of the R-tensor values from nonlinear least-squares simulations:*								
	*Buffer* *TOAC^x^ (x = 0, 2)* *TOAC^13^*	*R_x_ (5%), R_y_ (5%), R_z_ (5%)* *R_x_ (5%), R_y_ (2%), R_z_ (5%);*						
	*LPC micelles* *TOAC^0^* *TOAC^x^ (x = 2, 13)*	*Site 1: R_x_ (5%), R_y_ (5%), R_z_ (10%)* *Site 2: R_x_ (10%), R_y_ (2%), R_z_ (10%)* *R_x_ (5%), R_y_ (5%), R_z_ (10%);*						

In aqueous solution, the compounds display narrow lines (Figure 6A) as expected for small molecules tumbling in a non-viscous solvent. Nonlinear least-squares simulations showed that a symmetric Brownian model for the rotational diffusion tensor was capable of precisely capturing the fast mobility of the TOAC derivatives. As shown in Table V, the order of the spin label mobility in aqueous solution is [TOAC^13^]Ctx(Ile^21^)-Ha < [TOAC^2^]Ctx(Ile^21^)-Ha < [TOAC^0^]Ctx(Ile^21^)-Ha. The lowest mobility presented by TOAC^13^ derivative can be attributed to both the position of the nitroxide spin label in the peptide sequence and the acquisition of an α-helical structure as shown by our CD data.

Two additional parameters, which are usually used to get detailed information on folding and local contacts, can also be considered: the polarity, 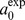, of the peptide environment surrounding the label as well as the peak-to-peak line width of the central line, *W_0_*. These parameters can be largely affected by the interaction of the spin label with neighboring backbone atoms or side chains of adjacent residues, for instance. As a consequence, the local contacts can impose a restricted, anisotropic motion to the spin probe [59]. In this case, the apparent hyperfine splitting decreases and the width of the central line of the ESR spectrum increases.

The 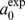
values obtained from the ESR spectra of TOAC^2^ and TOAC^13^ in aqueous solution showed a considerable reduction when compared to [TOAC^0^]Ctx(Ile^21^)-Ha (Table IV). This effect is most likely due to a shielding of the nitroxide radical from the water phase by the side chain of neighboring residues: Leu^3^ for the TOAC^2^ analogue and Ala^12^ and Phe^14^ for the TOAC^13^ derivative. Moreover, the striking increase of the line width (*W_0_*) of the [TOAC^13^]Ctx(Ile^21^)-Ha ESR spectrum compared to those of the other peptides (Table V) suggests a strong interaction of the spin probe with neighboring side chains and/or backbone atoms. These two effects can most likely be explained by the folding of the residues adjacent to TOAC in the [TOAC^13^]Ctx(Ile^21^)-Ha. Our ESR results thus indicate that the nitroxide radical and the neighboring residues in the [TOAC^13^]Ctx(Ile^21^)-Ha peptide adopted a more structured conformation in aqueous solution, which is in agreement with our CD results that indicated an α-helical conformation in aqueous solution (Fig. 3A).

ESR spectra of TOAC-labeled peptides bound to LPC micelles are shown in Figure 6B. In the membrane mimetic, where all the peptides adopt a well-ordered α-helix (Fig. 3C), the spectra displayed broader lines than in aqueous solution, thus corresponding to more immobilized spin label populations. This quite large broadening after addition of LPC cannot be explained only by the acquisition of an ordered secondary structure. In fact, considerable changes on the polarity (Table IV), line width, order parameters, and correlation times (Table V) indicate that the peptide directly interacts with the membrane mimetic, which is in agreement with our fluorescence assays. However, the results suggest that TOAC samples different environments at the lipid/water interface (e.g. total exposure to solvent, total immersion into the micelle hydrophobic core, and/or in between the polar head groups).

**Figure 7:**
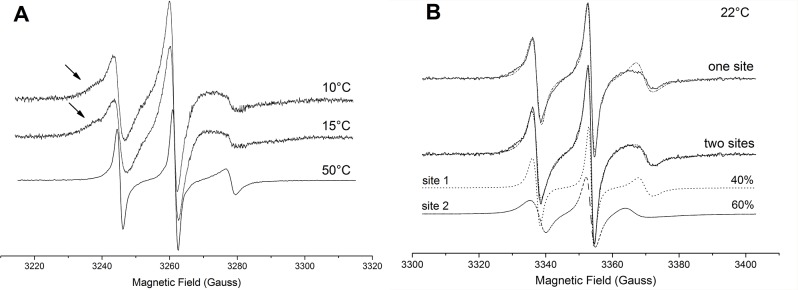
Representative ESR spectra acquired at 10, 15, and 50°C corresponding to a dynamic equilibrium between the membrane-bound and membrane-unbound N-terminal conformations (A). Note the heightening of the spin population experiencing a more restricted re-orientational motion at low temperatures. [TOAC^0^]Ctx(Ile^21^)-Ha ESR spectrum in LPC micelles (solid lines) acquired at 22°C and the best nonlinear least-squares fits (dashed lines) using one (top) or two (bottom) spectral components (B).

As shown by the arrows in Figure 6B, the shape of the low field resonance in the [TOAC^0^]Ctx(Ile^21^)-Ha ESR spectrum is not characteristic of a spin label residing in only one microenvironment. This two-peak feature might be either due to a simple partition of the peptide into the LPC micelle and the aqueous phase or due to two different conformations of its N-terminus. To gain more insights on the origin of these possible two spectral components, the temperature dependence of the TOAC ESR spectrum was recorded from 10 to 70°C and nonlinear least-squares simulations with one and two components were performed. Representative spectra at three temperatures are shown in Figure 7A. It is worth noting that the lower the temperature, the more evident the two-peak feature of the spectrum (arrows in Figure 7A). Figure 7B shows the 22°C [TOAC^0^]Ctx(Ile^21^)-Ha ESR spectrum that was best-fit with either a single- or a two-component theoretical spectrum. As can be observed, the single-component fit yielded an unsatisfactory result, with poor fitting of the spectrum at both low- and high-field lines.

The two-component nonlinear least-squares simulations indicate that the averaged correlation time of the more mobile component in the presence of the micelle at 22°C (0.84 ns) is more than five times slower than that obtained in solution (0.15 ns) at the same temperature. Moreover, we found that a weak orienting potential (*c*_20_ = 0.35, *S*_0_ = 0.07, Table V) imposed by the peptide conformation and/or the lipid head groups restricts the re-orientational motion of this spin population. This gives rise to an anisotropic distribution of orientations in which the spin label is able to move. The rotational diffusion of the mobile component is then described by an axial rather than by a symmetric tensor with rotational diffusion rates of 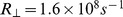 and 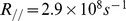. The second component was found to be much less mobile (<τ> = 3.3 ns) and more ordered (*S*_0_ = 0.14) than the previous one. So, these local structural and dynamics features cannot be explained by a simple partition of the peptide into the membrane mimetic environment and the water phase. In addition, the lack of spin-spin interactions on the ESR spectrum, which could arise from peptide oligomerization or aggregation, indicates negligible peptide-peptide interactions. Therefore, the two-peak feature is most likely due to two different N-terminal conformations of the membrane-bound [TOAC^0^]Ctx(Ile^21^)-Ha.

To further investigate the local spin-label environment, the polarity dependence of the magnetic parameters was analyzed with the calculated isotropic hyperfine splitting (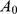) only, since the variations of *g_xx_* in the end of the fitting process were found to be very small. Table IV shows that the polarity of the more mobile component (16.60 G) was found to be similar to that presented by TOAC in aqueous solution (16.63 G for TOAC^0^ derivative). On the other hand, the second, more immobilized spin-label population presented an isotropic hyperfine coupling of 15.90 G. Although this value might be somehow contaminated by artifacts from slow motion, since the spin-label environmental polarity is only truly reflected on the fast motional regime [60], these results suggest that the immobilized spin population most likely represents an N-terminal conformation bound to the LPC micelle, whereas the mobile component is fully exposed to the water phase.

The ESR spectra of [TOAC^2^]Ctx(Ile^21^)-Ha and [TOAC^13^]Ctx(Ile^21^)-Ha in the presence of LPC micelles can be seen in Figure 6B. The main difference when compared to the spectrum of TOAC^0^ derivative in LPC is a much larger broadening of their single-component. This much more restricted motion of the nitroxide radical is consistent with the presence of a stable, highly ordered membrane-bound helix. Nonlinear least-squares simulations show that the Brownian dynamics of the spin label in both peptides is anisotropic with averaged correlation times of 12.0 ns for TOAC^2^ (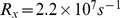,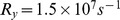,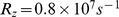) and 45.0 ns for TOAC^13^ (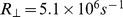 and 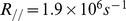) analogues (Table V). The polarity of the local spin-label environment was assessed by calculating the isotropic hyperfine coupling for these two peptide analogues from their ESR spectra acquired at 50°C according to Marsh *et. al* [61]. The 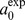
values obtained for TOAC^2^ (15.51 G) and for TOAC^13^ (15.05 G) derivatives are similar to those obtained from the dependence of the TOAC’s 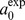
on membrane depth in fluid dipalmitoylphosphatidylcholine (DPPC) bilayers using the dipeptide Fmoc-TOAC-Aib-methoxy as a model system [62]. The isotropic hyperfine coupling for TOAC in this dipeptide predicts 15.64 G for the nitroxide radical inserted between carbons C3 and C6 of the *sn-2* lipid chain of DPPC and 15.05 G if it resides between C10 and C16. Thus, even though there still must be a slow motion contribution to the isotropic hyperfine coupling and also considering that the dependence of TOAC’s 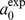
on depth in LPC micelles must be somewhat different from that determined for this spin label inserted in fluid DPPC, it is clear that TOAC is fully inserted into the LPC micelle in these two analogues. That is, TOAC, incorporated at position 2, most likely resides in the apolar environment of the LPC micelle, but close to the polar-apolar interface, whereas when incorporated at position 13 lies deeper in the hydrophobic core of the membrane mimetic environment.

**Figure 8:**
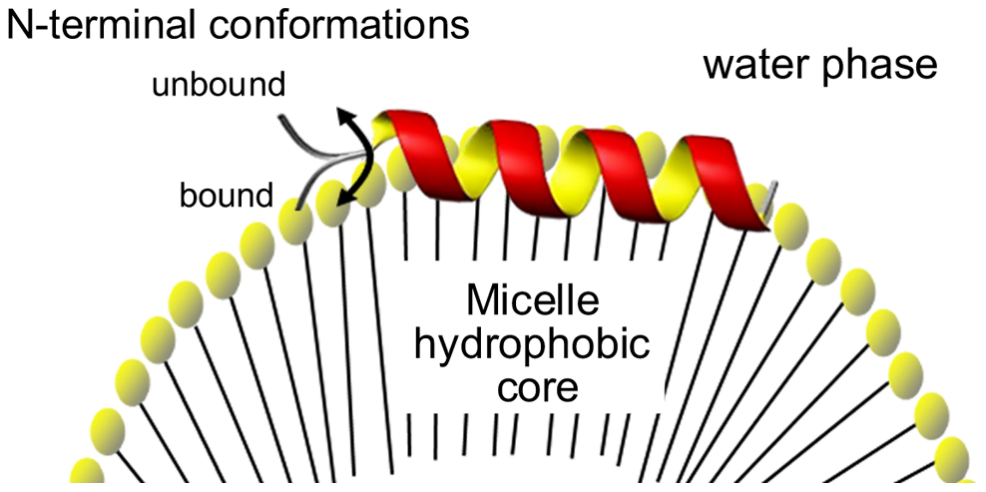
Schematic representation of the Ctx(Ile^21^)-Ha topology determined by ESR and nonlinear least-squares simulations showing the two N-terminal conformations: the immobilized, ordered membrane-bound state and the mobile, disordered form, fully exposed to the water phase.

Finally, considering that the LPC’s aggregation number is 139 [63], we have roughly one peptide per micelle in our experiments ([LPC] = 10 mmol L^−1^ and [peptide] = 80 µmol L^−1^). Independent of the mechanism of action (pore formation by barrel-stave, toroidal or carpet like), a peptide needs to reach a high concentration at a certain area of the membrane in order to form a transmembrane pore. According to this assumption and due to the low peptide:micelle molar ratio used in our assays, we can exclude peptide-peptide interactions, i.e. peptide aggregation or oligomerization, as discussed previously. So, the data obtained in the present study correspond to the initial interaction between the peptide and the micelle, immediately before what would be the pore-forming state in a lipid bilayer system. There is a consensus, independent of the pore-formation mechanism, that the polar face of the peptide is in contact with the aqueous medium whereas the apolar face is directed towards the hydrophobic core of the membrane (Figure 8) [64], [65].

### Conclusions

The present study showed that the ESR methodology based on the use of the TOAC spin probe is very sensitive to peptide local microenvironment and backbone dynamics. Furthermore, its use along with other spectroscopy techniques, such as reported here, can provide information about topology and mobility of the peptide in membrane mimetic environments. Our findings allowed the description of the peptide topology in LPC micelles, where the paramagnetic amino acid TOAC in all peptide derivatives samples different environments at the lipid/water interface. The N-terminal position was found to be in a dynamic equilibrium between an immobilized, ordered conformation in direct contact with the micelle surface, and a dynamically mobile disordered form, exposed to the water phase; on the other hand, TOAC at position 2 is experiencing the hydrophobic core of the micelle, but close to the membrane/water interface whereas this spin probe at position 13 is fully inserted into the membrane mimetic.
